# Supplementary material for: Identification of anisomycin, prodigiosin and obatoclax as compounds with broad-spectrum anti-parasitic activity
Source: PLoS Negl Trop Dis. 2020 Mar 20;14(3):e0008150. doi: 10.1371/journal.pntd.0008150 (PMC7112225; doi:10.1371/journal.pntd.0008150)
Supplement: Attachment — Submitted filename: Ehrenkaufer Multiple parasite PLoSNTD Response SUBMIT.docx [file pntd.0008150.s002.docx]

Stanford University School of Medicine

DIVISION OF INFECTIOUS DISEASES AND GEOGRAPHIC MEDICINE

S-143 GRANT BUILDING

300 PASTEUR DRIVE

Stanford, California 94305-5107

Upinder Singh, MD	TEL: 650.723.4045

Professor	FAX: 650 724. 3892

Division Chief, Infectious Diseases and Geographic Medicine	Email: usingh@stanford.edu

Department of Internal Medicine

February 14, 2020

Dear Editor,

We thank the reviewer’s input on the manuscript “Identification of anisomycin, prodigiosin and obatoclax as compounds with broad-spectrum anti-parasitic activity” and feel that their evaluation has been very helpful and improved the manuscript.

The responses to the comments are listed below.

**Reviewer 1:**

**Comment:** Reviewer #1: The results are clearly  presented in tables, figures, and text. While the results vary from drug to drug and organism to organism, enough of them are promising to make the study of interest and enough results are negative to assure that investigators were honest.

**Response:** We are grateful for the positive comments.

**Comment:** in the abstract line 54 and discussion line 424 they should be careful to say that there are no treatments for brain infection with Acanthamoeba, although there are available topical treatments for eye infections.

**Response:** Yes, this is a good point and information about topical eye treatment has been added to the discussion.

**Comment:** Reviewer #1: The goal here was to test four repurposed drugs that are effective versus Entamoeba in vitro versus other pathogenic protists. In each case the investigators, which are experts in each organism tested and from numerous universities, use assays that allow them to determine the EC50 for each compound.

**Response:** We appreciate the positive comments of the reviewer.

**Reviewer 2:**

**Comment:** Reviewer #2: The results are clearly presented and well written.  The results show they have much promise, in vitro, for rapid action against E. histolytica, though somewhat disappointing in the cidal washout experiment.  Some of the drugs have activity against other of the panel of parasites tested, though the only in vivo study was a negative study with Prodigiosin in C. parvum infection of NOD/SCID/gammaKO mice.  Despite this negative study, there is abundant information to justify rapid publication of the results.

**Response:** We appreciate that the reviewer feels there are enough data to justify publication of the results.

**Comment:**  A previous screen of the ReFrame compound library against Entamoeba histolytica led to the discovery of 4 compounds, Prodigiosin, Obatoclax, Anisomycin, and Nithiamide, that have previously been in human trials or use.  The authors report on advanced testing of these 4 compounds on advanced tests for E. histolytica bioactivity and to investigate a panel of other parasites including pathogenic free-living amoeba, cryptosporidium, plasmodium, and Schistosoma mansoni. The study design is appropriate to shed light on the broad activity of some of these compounds. I had no concerns about the methods

**Response:** We appreciate these comments.

**Comment:**  The figures are of good quality, except in the figure legend 4, it states we should see dots representing the values of the 4 animals tested but I can only see a very few dots.

**Response:** Thank you for making this comment. We have made the dots larger. Of note, some of the data points are overlapping each other, so it seems like there are only 3 animals, although the data are from 4 animals.

**Comment:** On Page 22 of the PDF, line 309, there is a reference to Table S1, but the Table they are referring to in line 309 should actually be Table 1.  Furthermore Table S1 should be moved down towards the end of the paper where it is discussed.

**Response:** Yes, we have changed this reference and moved Table S1.

**Comment:** Reviewer #2: The conclusions are reasonably well supported by the data but could be improved by my suggestions below. The relevance to advancing the field and therapies are discussed as well as (indirectly) the public health relevance.  I feel the paper's findings' applicability and promise would be way easier to evaluate if the authors made a comparative table of the 4 drugs, especially including their pharmacokinetic (PK) parameters.  The PK should include, where ever available:  plasma peak (Cmax) and exposure (AUC) information after various doses when give by oral (and IV) forms. This could be only the human data or better yet, include major efficacy species, such as mice.   The plasma protein binding of each drug would be useful information as well as oral bioavailability and CNS penetration.  Some of this data is (nonquantitatively) mentioned in passing, and is probably available in the public domain.  Then the discussion could be expanded into whether higher or lower doses that have previously been given to humans would likely be necessary, or even whether intrathecal delivery for the amoebic encephalitis cases might be necessary.

**Response:** Thank you for the comments. We have made a table (Table 2) with data that we found in the literature and added discussion of human dosing levels where applicable to the text. We have also discussed the possibility of intrathecal delivery in the discussion.

**Comment:** The above data might further inform the negative study on C. parvum and expand the discussion here.  For instance is prodigiosin so well absorbed that it might not be delivered to the terminal ileum/proximal colon where the C. parvum infection is highest.

**Response:** Thank you for the suggestion. We have added some comments on this possibility to the discussion.

**Comment:** The host cell toxicity data is only shown for one mammalian cell line, HepG2, and is certainly alarming for Anisomycin, Prodigiosin, and Obatoclax where the CC50 values for HepG2 are well below what is considered efficacious for many parasites.  The authors don't discuss this data, but I feel they should. Perhaps that data is mitigated by the sheer fact that these therapies have been in humans already, but it's all relative to the levels achieved in vivo and the serum protein binding of the compounds (see #1 above).

**Response:** This is a good suggestion. The data has been compiled from the literature and added to Table 2

We appreciate the ability to respond to the comments and concerns. All authors have read the paper and agree with its contents. Please do not hesitate to contact me if you need further information.

Sincerely,

Upinder Singh, MD

Professor and Division Chief, Infectious Diseases and Geographic Medicine

Department of Internal Medicine

Stanford University School of Medicine

Tel: 650-723-4045; Email: [usingh@stanford.edu](mailto:usingh@stanford.edu)
